# Experimental Investigation on the Fracture Behavior of Basalt Fiber-Reinforced Shotcrete

**DOI:** 10.3390/ma19050842

**Published:** 2026-02-24

**Authors:** Junbo Guo, Wei Shi, Kun Wang, Lingze Li, Dingjun Xiao

**Affiliations:** 1 The Third Engineering Bureau Co., Ltd. of China An’neng Construction Group, Chengdu 611130, China; 2 Department of Mining Engineering, School of Environment and Resources, University of Science and Technology Southwest, Mianyang 621010, China

**Keywords:** basalt fiber-reinforced concrete, freeze–thaw cycles, dynamic and static fracture behavior, brazilian disk test, crack propagation

## Abstract

Basalt fiber-reinforced concrete is increasingly being used in shotcrete support systems for rock mass excavation engineering due to its superior mechanical properties and durability. Rapid freeze–thaw cycling tests were performed to simulate freeze–thaw conditions in order to meticulously investigate the dynamic and static fracture behaviors of basalt fiber-reinforced concrete in freeze–thaw environments. Then, utilizing a Split Hopkinson Pressure Bar (SHPB) system and rock testing equipment, dynamic and static fracture tests were performed on developed Mode I, mixed-mode I/II, and Mode II platform Brazilian disk specimens. Under freeze–thaw conditions, the dynamic and static fracture propagation velocities of specimens with diverse crack propagation modes were determined. Based on this, LS-DYNA numerical simulations were used to perform inverse evaluations of crack propagation processes in specimens with varied fracture modes and Mode I fracture specimens with variable basalt fiber contents. We were able to calculate the effective stress field distributions during crack propagation with dynamic loading. The data indicate that different fracture modes present significantly distinct crack propagation issues. Pure Mode I fracture specimens exhibit the most straightforward crack propagation, with a maximum effective stress of roughly 25 MPa after crack penetration. With a maximum effective stress of around 31 MPa following crack penetration, the mixed-mode I/II fracture specimens exhibit considerable propagation difficulties. Mode II fracture specimens are the hardest to propagate after crack penetration because of their maximum effective stress of 64 MPa. Additionally, the optimal basalt fiber content was determined to be in the range of 0.35% to 0.45%, at which the concrete exhibited the best fracture toughness and freeze–thaw resistance. Furthermore, the evolution characteristics of the displacement of the crack tip opening under different fracture modes are revealed. A theoretical basis for stability analysis and design of excavation engineering structures under dynamic stress and associated freeze–thaw conditions is provided by the study’s findings.

## 1. Introduction

Basalt fiber, a new inorganic nonmetallic reinforcing material, has recently emerged as a research hotspot for improving shotcrete’s overall performance due to its high specific strength, exceptional resistance to chemical corrosion, and good interfacial compatibility with cementitious matrices [1,2,3,4,5,6]. Numerous experiments have shown that adding basalt fibers to concrete can successfully prevent microcracks from starting and spreading, encouraging a change in the failure mode from brittle to ductile. This not only greatly increases the material’s resistance to impact and cracking, but it also doubles its fatigue life. Furthermore, basalt fiber-reinforced concrete continues to show exceptional resilience in challenging environmental circumstances such as salt spray, freeze–thaw cycles, and exposure to acidic or alkaline environments [7,8,9,10].

Concrete constructions are frequently subjected to freeze–thaw cycles during extended service periods in northern China and other high-altitude cold climates. Concrete’s mechanical qualities frequently deteriorate over time due to the phase shift in pore water and the resulting harm to the interior microstructure during freeze–thaw operations. The likelihood of unstable fracture propagation increases dramatically when such buildings are subjected to additional dynamic stresses caused by engineering blasting, earthquakes, or traffic vibrations. This might easily result in safety incidents [11,12,13,14,15,16,17]. The development of mechanical properties of concrete materials in cold climates during freeze–thaw cycles must be thoroughly examined in order to guarantee the long-term safe operation of engineering constructions. Previous research has demonstrated that adding fibers to concrete may significantly improve its mechanical performance. Because of its abundant raw material resources, superior resistance to acid and alkali corrosion, high temperature resistance, advantageous tensile properties, and cost-effectiveness, basalt fiber has become a green and environmentally friendly reinforcing material with a wide range of potential applications [18,19,20].

Despite these advances, several research gaps remain unaddressed. Most existing studies have focused on the macroscopic mechanical degradation of fiber-reinforced concrete under freeze–thaw conditions, while systematic investigations into the dynamic and static fracture behaviors, particularly the crack propagation mechanisms under different fracture modes (Mode I, mixed-mode I/II, and Mode II), are still lacking. Furthermore, the evolution of stress fields during crack propagation and the optimal basalt fiber content range for maximizing fracture toughness and freeze–thaw resistance have not been thoroughly explored.

To address these gaps, the primary objectives of this study are as follows: (1) to systematically investigate the dynamic and static fracture characteristics of basalt fiber-reinforced concrete under freeze–thaw cycles; (2) to reveal the influence of fracture modes on crack propagation velocity and stress field evolution; and (3) to determine the optimal basalt fiber content range for enhancing fracture toughness and freeze–thaw durability.

Central straight-cracked flattened Brazilian disk (CSTFBD) specimens with three distinct crack inclination angles were created based on the aforementioned study background, and experimental studies were conducted under various numbers of freeze–thaw cycles [21,22,23]. The fracture propagation velocity under various test circumstances was measured using a crack propagation gauge (CPG). The fracture toughness of the specimens under various levels of freeze–thaw damage was ascertained by combining the quasi-static approach with an experimental–numerical coupled analysis. The effects of the freeze–thaw environment and crack parameters on the material’s fracture properties were methodically examined from the viewpoints of fracture toughness and crack propagation behavior. Additionally, the fracture mechanism of basalt fiber-reinforced shotcrete under freeze–thaw conditions was further clarified by using ANSYS/LS-DYNA2019R3 to numerically simulate the stress field development during crack propagation.

The novelty of this study lies in three aspects: (1) it provides the first systematic comparison of dynamic and static crack propagation behaviors of basalt fiber-reinforced concrete under Mode I, mixed-mode I/II, and Mode II fracture conditions in freeze–thaw environments; (2) it combines SHPB experiments with LS-DYNA numerical simulations to reveal the evolution characteristics of the effective stress field during crack propagation; and (3) it identifies the optimal basalt fiber content range and quantifies its improvement effects on fracture toughness and freeze–thaw resistance, offering practical guidance for shotcrete engineering in cold regions.

## 2. Specimen Preparation and Test Procedure

### 2.1. Preparation of BFRC Fracture Characteristic Test Specimens

(1) Design of Specimen Configuration

**Figure 1:**
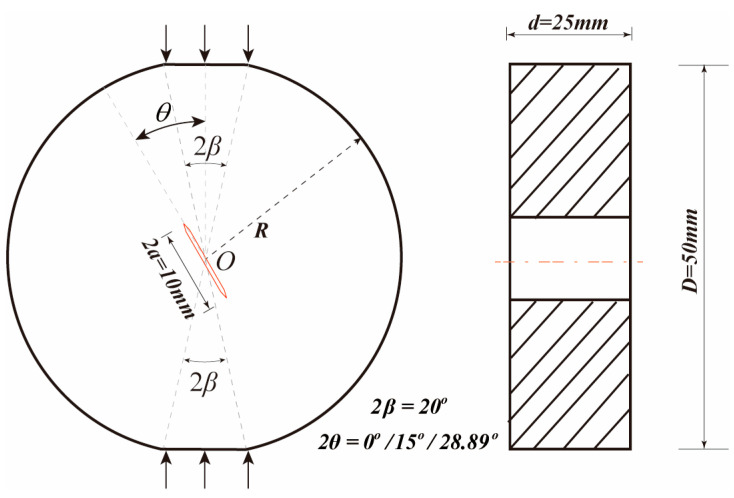
Flattened Brazilian disk specimen.

The specimen arrangement used in this work to investigate the fracture properties of basalt fiber-reinforced concrete (BFRC) is illustrated in Figure 1. Central straight-through flattened Brazilian disk (CSTFBD) samples were employed [24]. The specimen measured φ50 mm × 25 mm, with a disk diameter of 50 mm and a thickness of 25 mm. Each specimen had a constructed straight-through central fracture with a length of 2*a* = 10 mm. The relative fracture length was calculated using the formula α = 2*a*/*D* = 0.2, where *D* is the specimen’s diameter.

According to prior research [25,26,27,28], flattened loading platforms were machined on the upper and lower ends of the specimen, and varied fracture modes may be obtained by altering the platform loading angle, which controls the stress condition at the crack tip. To ensure the comparability and consistency of test findings under varied settings, all specimens were developed as CSTFBD specimens with the same relative crack length. When the loading angle reaches a critical value, it is possible to achieve pure Mode II fracture propagation. Equation (1) expresses the link between the critical loading angle and the prefabricated crack inclination angle [29,30]:

(1) $$\left\{\begin{array}{l} \theta =30.982-6.6667\alpha -19.048\alpha^{2}\\ 0.1<\alpha <0.9 \end{array}\right.$$

The loading angle for a pure Mode II fracture in this investigation was determined to be $\theta_{ΙΙ}$ based on theoretical analysis and findings. To represent the mixed-mode I/II fracture configuration, an intermediate loading angle ($\theta_{Ι/ΙΙ}$) was chosen between the pure Mode I and pure Mode II loading angles, whereas $\theta_{ΙΙ}$ represents the pure Mode I fracture mode. This combination of loading angles allowed for a systematic analysis of crack propagation characteristics in pure Mode I, mixed-mode I/II, and pure Mode II fracture modes.

(2) Mix proportion design of basalt fiber-reinforced concrete

The basalt fiber-reinforced concrete (BFRC) employed in this investigation has a target strength grade of C30. The concrete mixture was made up of water, cement, fine aggregate, coarse aggregate, and basalt fibers in the required quantities.

Ordinary Portland cement (P.O 42.5) was chosen as the cementitious ingredient to achieve consistent strength development. Natural river sand with a fineness modulus of 2.5 was employed as the fine aggregate, resulting in a well-graded particle size distribution and high homogeneity in the fresh concrete. Natural rounded gravel with particle sizes ranging from 4 to 6 mm was used as the coarse aggregate to meet the molding requirements of small-sized specimens and limit the impact of aggregate size on mechanical qualities. Table 1 lists the key physical and mechanical parameters of short-cut basalt fibers used to strengthen the cement matrix during concrete production.

Basalt fiber-reinforced concrete specimens meeting the experimental requirements were successfully prepared using the aforementioned raw material selection and mix proportion design, providing a stable and reproducible material basis for subsequent freeze–thaw cycling treatments and dynamic and static fracture mechanics tests.

Based on multiple prior studies [31,32,33], a basalt fiber volume fraction of 0.15% was chosen as the test condition for this investigation. Previous research has shown that this dosage level is both reasonable and practically applicable, as it can significantly improve the mechanical properties and crack resistance of concrete while avoiding the reduction in workability and increase in material cost associated with higher fiber contents, resulting in a favorable overall performance.

Under this fiber content condition, Table 2 summarizes the proportional mass proportions of the basic materials used to make basalt fiber-reinforced concrete.

### 2.2. Experimental Procedure for Dynamic and Static Fracture Testing Under Freeze–Thaw Conditions

**Figure 2:**
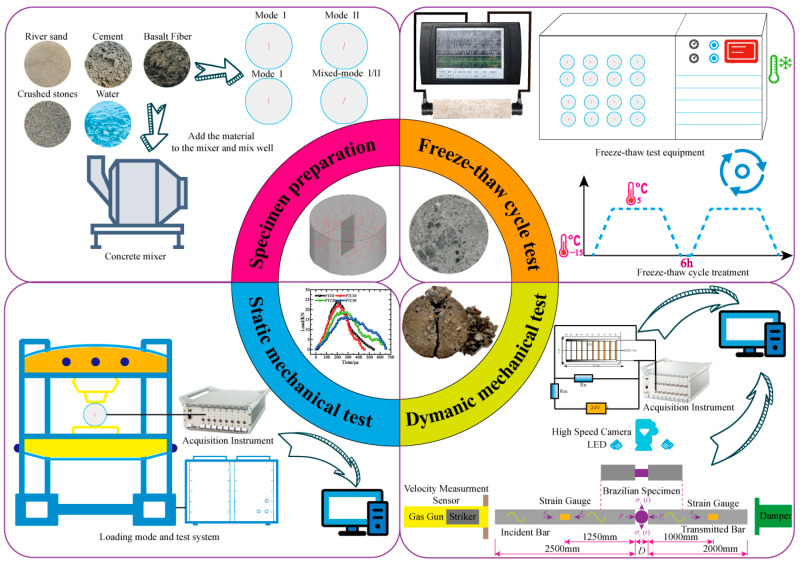
Experimental procedure.

Figure 2 illustrates the experimental approach used in this investigation. The whole procedure is organized clockwise and consists of four major stages: specimen preparation, freeze–thaw cycling and performance testing, dynamic loading tests, and static loading tests. Each stage is linked and carried out sequentially to illustrate the progression of fracture behavior in specimens exposed to freeze–thaw conditions.

The process starts with specimen preparation. Three types of central straight-through flattened Brazilian disk (CSTFBD) specimens with different pre-crack inclinations are manufactured using a predetermined mix proportion and geometric parameters to ensure consistency and comparability in geometric configuration and initial defect conditions. The specimens are then cured under regular circumstances until the desired curing age is attained, reducing the impact of material variability on the experimental results.

After curing, the specimens are freeze–thawed to imitate the recurrent temperature fluctuations that concrete buildings encounter in cold climates. The freeze–thaw temperature range is 5 °C to −15 °C (16 h freezing at −15 °C, 8 h thawing at +5 °C), with 0, 10, 20, and 30 cycles chosen to represent varying amounts of freeze–thaw damage. This environmental simulation applies to all three pre-crack inclination types. By varying the number of freeze–thaw cycles, the study analyzes the impacts of freeze–thaw action on internal damage progression and fracture performance deterioration [34].

After the freeze–thaw procedure, dynamic and static loading tests are performed. Dynamic loading studies are used to investigate crack initiation and propagation under freeze–thaw damage circumstances, whereas static loading tests are used to quantify critical fracture mechanics characteristics, such as fracture toughness, under different freeze–thaw situations. By comparing results from different freeze–thaw cycles and pre-crack inclinations, the combined effects of the freeze–thaw environment and crack geometry on fracture behavior are examined.

This experimental procedure’s systematic execution incorporates freeze–thaw simulation, crack propagation observation, and fracture parameter acquisition, resulting in a credible experimental foundation for further fracture mechanism research and numerical simulation investigations.

**Figure 3:**
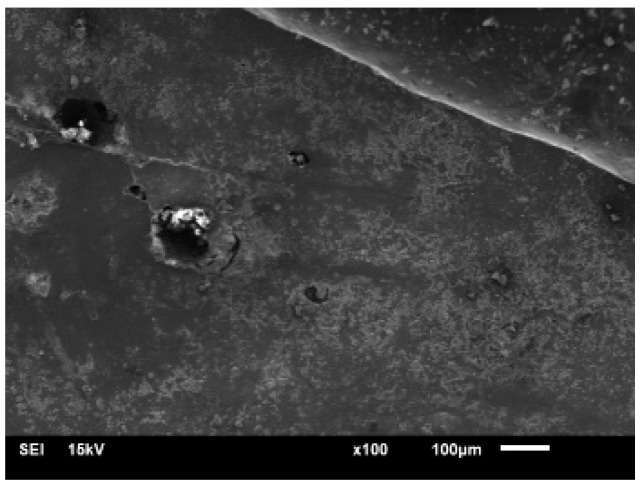
SEM images of fiber-reinforced concrete.

To compare the fracture reaction of basalt fiber-reinforced concrete (BFRC) under various stress situations, static fracture reference tests were performed using universal testing equipment. The experiments used a displacement-controlled loading technique with a rate of 0.5 mm/min to assure stability and reliable data gathering. Table 3 summarizes the individual test circumstances and parameter settings. The concrete slump test shows that the plain concrete has a slump of 180 mm and a uniaxial compressive strength of 28–29 MPa, while the basalt fiber-reinforced concrete has a slump of 140–160 mm. The dispersion of fibers in the cementitious matrix was examined by SEM observation of fracture surfaces. As shown in Figure 3, the fibers are uniformly distributed without noticeable agglomeration. Image analysis results indicate that the coefficient of variation for fiber spacing is less than 10%, confirming satisfactory fiber dispersion quality.

The dynamic fracture tests were carried out with a Split Hopkinson Pressure Bar (SHPB) system. Controlling the projectile launch pressure stabilized the impact velocity at 5 m/s, resulting in consistent dynamic loading. Table 4 summarizes the related test conditions and loading parameters.

#### 2.2.1. Determination of Material Parameters for BFRC Specimens Under Freeze–Thaw Conditions

(1) Testing equipment and method

While casting the basalt fiber-reinforced concrete (BFRC) specimens, an extra batch of conventional prismatic specimens measuring 100 mm × 100 mm × 400 mm was created concurrently. The longitudinal and shear wave velocities of BFRC specimens were measured using an RSM-SY5 ultrasonic testing system following several freeze–thaw cycles, resulting in the typical longitudinal wave velocity $C_{p}$ and shear wave velocity $C_{s}$.

(2) Calculation of dynamic Poisson’s ratio and dynamic elastic modulus

The material’s dynamic mechanical properties, such as the dynamic Poisson’s ratio $\mu_{d}$, were calculated using elastic wave propagation theory and observed longitudinal and shear wave velocities. The dynamic elastic modulus is $E_{d}$. This method allowed for a systematic measurement of the dynamic mechanical properties of BFRC under varied freeze–thaw conditions, giving a valid foundation for selecting constitutive models and allocating material parameters in future numerical simulations. Table 5 summarizes the computed dynamic mechanical characteristics.

(2) $$\left\{\begin{array}{l} \mu_{d}=\frac{\left(C_{p}^{2}-2C_{s}^{2}\right)}{2(C_{p}^{2}-C_{s}^{2})}\\ E_{d}=\frac{\rho C_{s}^{2}(3C_{p}^{2}-4C_{s}^{2})}{C_{p}^{2}-C_{s}^{2}} \end{array}\right.$$

#### 2.2.2. Determining Crack Propagation Velocity

**Figure 4:**
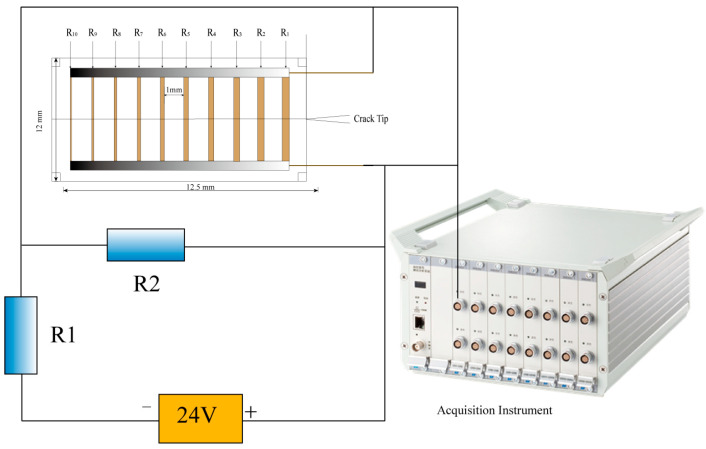
Experimental principle schematic diagram and physical object diagram.

In this work, a custom-made crack propagation gauge (CPG) from Shenzhen Microelectronics Technology Co., Ltd., was used to assess the crack propagation velocity of basalt fiber-reinforced concrete (BFRC) specimens after various numbers of freeze–thaw cycles. The CPG model utilized was the BKX5-10CY with a starting resistance of 5 Ω. Figure 4 depicts both an image of the CPG used in the experiment and a schematic diagram demonstrating its operation [35,36,37,38].

The CPG is 12.5 mm in length and 12 mm in width. It consists of 10 parallel resistance wires with a regular gap of 1 mm between them. The essential operating concept is as follows: when a fracture propagates through the specimen, the resistance wires are successively severed, resulting in gradual resistance variations. Real-time recording and analysis of resistance variation signals allows for exact determination of the crack’s temporal history and propagation velocity.

The DH5960 ultra-dynamic signal acquisition system was employed to supply the bridge voltage with a constant voltage source of 24 V, ensuring optimal CPG operation and dependable signal capture. In addition, two safety resistors for current limiting and distribution were added to the measuring circuit to provide a consistent current output and avoid CPG damage caused by high power. To restrict the main circuit current, resistor R1 (500 Ω) was connected in series with the CPG. Resistor R2 (50 Ω) was connected in parallel to stabilize current distribution and promote measurement safety. This circuit arrangement efficiently guaranteed the precision and reliability of CPG readings during dynamic fracture testing.

**Figure 5:**
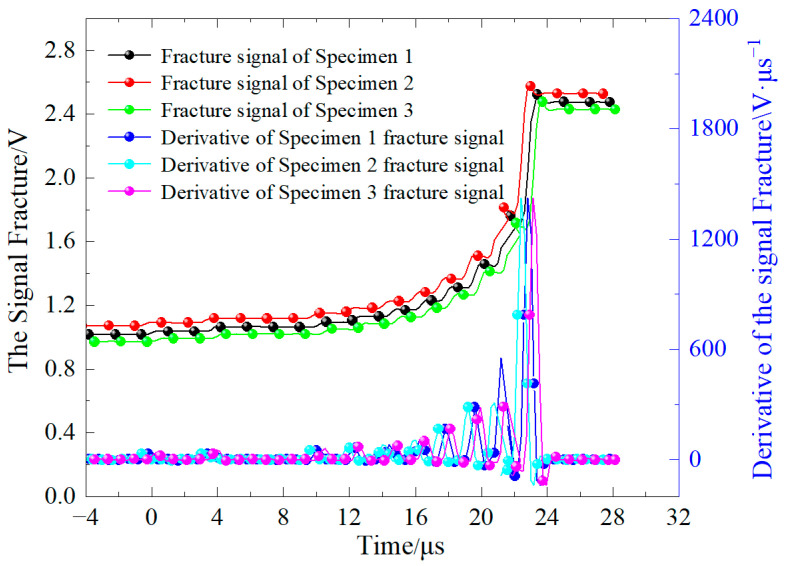
The curve of signal fracture versus time.

Using the D-30-15 condition as an example, Figure 5 depicts the voltage–time response curve of the crack propagation gauge (CPG) at the crack tip of the basalt fiber-reinforced concrete (BFRC) specimen during the dynamic impact test, as well as its time derivative. The CPG voltage signal shows a definite stepwise rise overall. This behavior implies that as the fracture progresses through the specimen, the crack front gradually severs the resistance wires within the CPG, resulting in discrete jumps in the voltage signal.

Furthermore, by differentiating the voltage signal with respect to time, a number of distinct peaks may be discovered. Each peak corresponds to the exact instant when a resistance wire in the CPG is severed by the fracture. Using the time intervals between subsequent wire failures and the known spacing between the resistance wires, the instantaneous crack propagation velocity and development under dynamic stress may be statistically determined. This approach efficiently describes the temporal aspects and dynamic behavior of crack propagation, providing a solid experimental foundation for studying the dynamic fracture properties of BFRC under various freeze–thaw cycle settings. Tests on three replicate specimens under identical conditions indicate that the time measurement error is within 1–2 μs.

## 3. Experimental Results and Analysis

### 3.1. Determination of Dynamic and Static Loading

Using one-dimensional stress wave theory, the load given to the CSTFBD specimen by the incidence bar, *P_i_(t)*, may be calculated by superimposing the incident and reflected wave signals. The transmitted wave signal may be used to calculate the load communicated by the transmission bar to the specimen (*P_t_(t)*). The related calculation formulae are given below:

(3) $$P_{i}\left(t\right)=E_{b}A_{b}\left[\varepsilon_{i}\left(t\right)+\varepsilon_{r}\left(t\right)\right]$$

(4) $$P_{t}\left(t\right)=E_{b}A_{b}\varepsilon_{t}\left(t\right)$$

Thus, the specimen is subjected to an average load throughout the operation $\bar{P}(t)$:

(5) $$\bar{P}\left(t\right)=\frac{P_{i}\left(t\right)+P_{t}\left(t\right)}{2}$$

**Figure 6:**
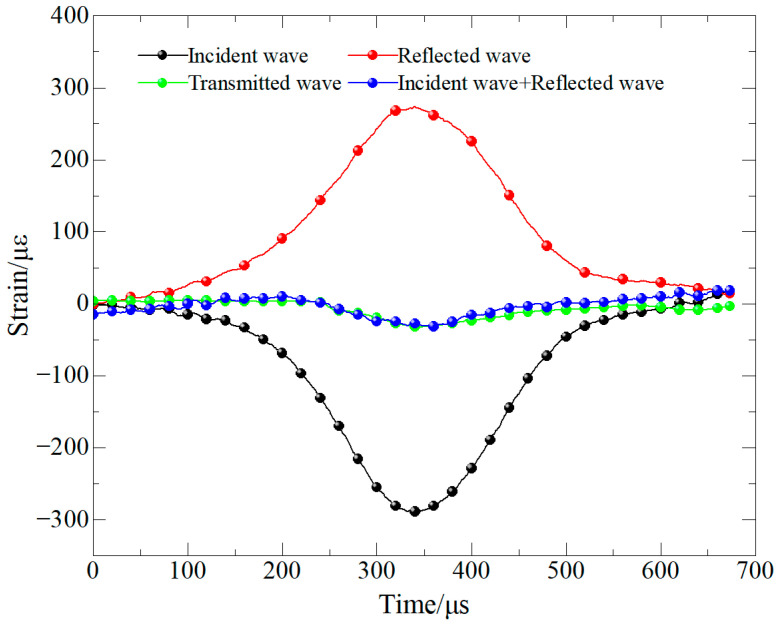
Stress equilibrium verification for dynamic splitting tensile tests.

We have verified the stress equilibrium condition of the SHPB tests by comparing the waveforms of incident + reflected waves and the transmitted wave, as show Figure 6. The results show that the two waveforms closely coincide during the main loading stage, satisfying the validity requirements for dynamic testing.

**Figure 7:**
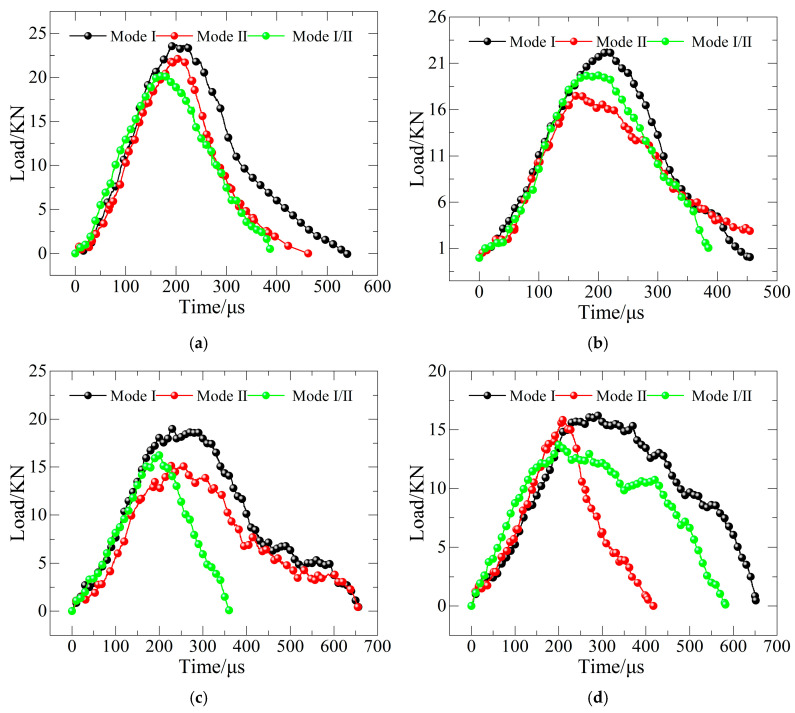
Dynamic load–time curves of concrete reinforced with basalt fiber: (**a**) FTCs 0; (**b**) FTCs 10; (**c**) FTCs 20; and (**d**) FTCs 30.

The specimens’ dynamic loading was computed theoretically using the given formulae. Figure 7 depicts the dynamic load–time curves for specimens with three different pre-crack angles throughout the test’s freeze–thaw cycles.

**Figure 8:**
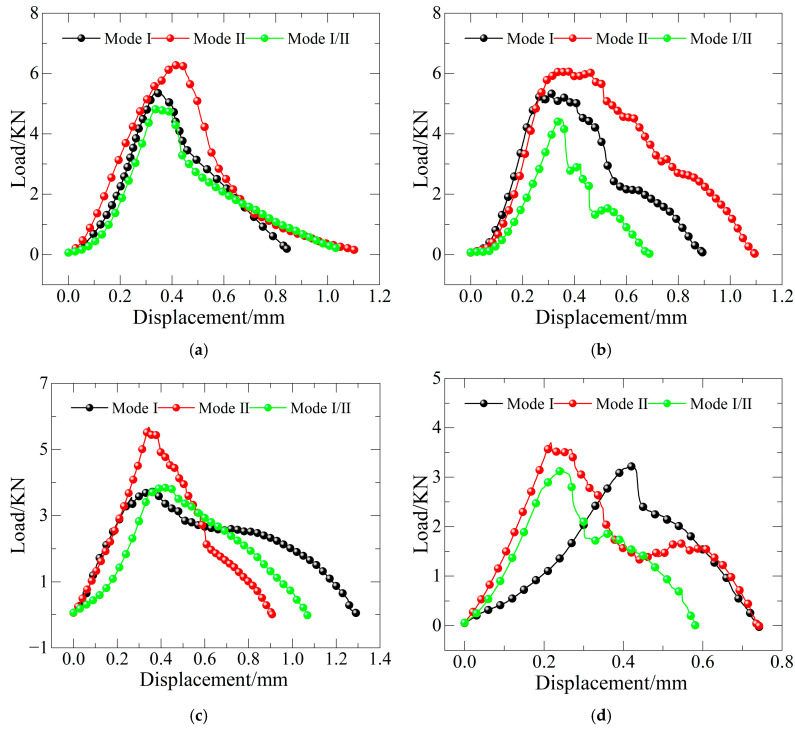
Static load–displacement curves of concrete reinforced with basalt fiber: (**a**) FTCs 0; (**b**) FTCs 10; (**c**) FTCs 20; and (**d**) FTCs 30.

The static loads of the specimens were represented by the peak loads recorded from the universal testing machine. The load–displacement curves under different static test conditions are shown in Figure 8.

### 3.2. Effect of Freeze–Thaw Cycles on Crack Propagation Under Different Fracture Modes

#### 3.2.1. Evaluation of the Velocity of Crack Propagation Under Dynamic Loading

(1) Evaluation of Mode I Pre-Cracked Specimens’ Crack Propagation Velocity

**Figure 9:**
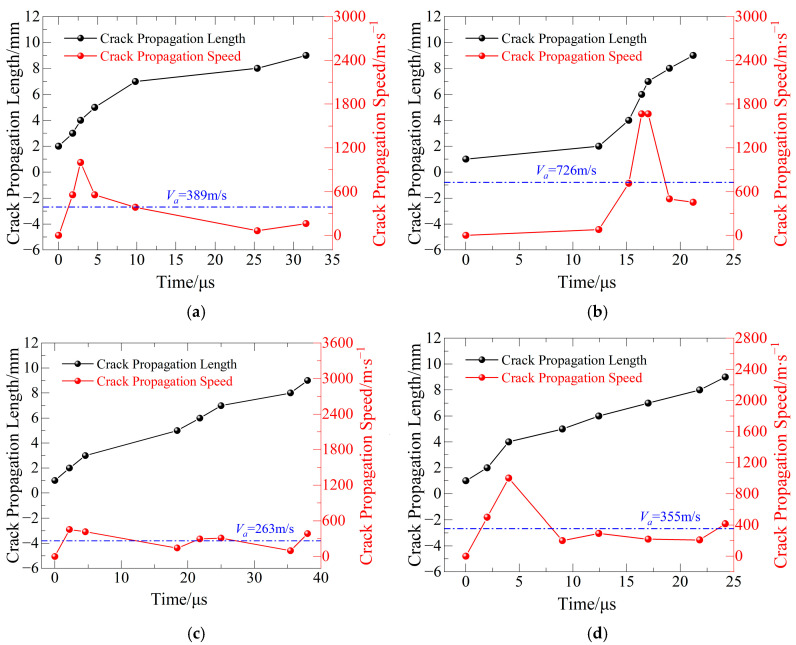
Crack propagation length/velocity and time relationship for Mode I specimens: (**a**) FTCs 0; (**b**) FTCs 10; (**c**) FTCs 20; and (**d**) FTCs 30.

The fracture tip position and accompanying crack propagation velocity of basalt fiber-reinforced concrete under various freeze–thaw cycle counts at a loading angle of *θ* = 0° are shown in Figure 9. The specimens primarily show pure Mode I fracture at this loading angle, with fractures starting steadily from the constructed crack and moving forward in the original path, and the minimum, maximum, and average propagation velocities are shown in Table 6.

The Figure shows that, for the first three test groups, the fracture propagation velocity gradually rises with the number of freeze–thaw cycles. This pattern suggests that freeze–thaw action causes severe damage of the interior microstructure of basalt fiber-reinforced concrete. The enlargement of pores and the buildup of microcracks over repeated freeze–thaw cycles reduce the material’s total crack resistance, allowing for quicker fracture propagation under external force.

In contrast, the fourth test group has a clear blank spot in the fracture propagation velocity data, which is accompanied by a decreased average crack propagation velocity. This phenomenon is most likely caused by the presence of massive aggregates dispersed throughout the fracture propagation route. When the advancing crack contacts coarse aggregates, the propagation channel may be diverted or momentarily obstructed, resulting in a localized drop in crack propagation rate and, in certain situations, making it impossible for the CPG to properly capture the fracture within a short time period. As a result, it can be concluded that coarse aggregates have a blocking and bridging impact on fracture propagation, which is the major cause of the lower average crack propagation velocity seen in this sample group.

(2) Crack Propagation Velocity Analysis in Mixed-Mode I/II Prefabricated Cracks

**Figure 10:**
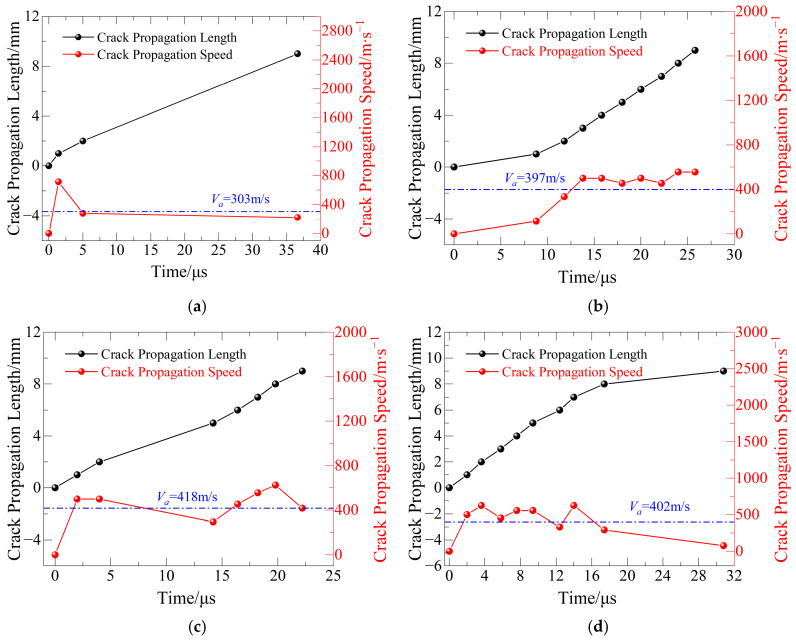
Crack propagation length/velocity and time relationship for Mode I/II mixed-mode specimens: (**a**) FTCs 0; (**b**) FTCs 10; (**c**) FTCs 20; and (**d**) FTCs 30.

Figure 10 shows how fracture tip position and propagation velocity of basalt fiber-reinforced concrete (BFRC) vary with the number of freeze–thaw cycles and loading angle *θ* = 15°. Under this loading scenario, the specimens display mixed-mode I/II fracture, which occurs when tensile and shear forces act together to initiate and propagate cracks. As a result, the crack propagation route is more complicated than that found in pure Mode I fracture; the minimum, maximum, and average propagation velocities are shown in Table 7.

When compared to the loading angle for pure Mode I fracture (*θ* = 0°), the crack propagation direction in mixed-mode I/II circumstances deviates substantially from the prefabricated crack’s axis. The fracture route exhibits significant bending and irregularity, reducing the predictability and stability of the crack propagation process. As a result, the interaction between the approaching crack front and the resistance wires of the crack propagation gauge (CPG) becomes more unpredictable, resulting in less effective signals collected by the CPG.

Nonetheless, the available effective data points clearly show that the crack propagation velocity increases in an overall oscillatory pattern with the number of freeze–thaw cycles. This discovery shows that, even under mixed-mode I/II fracture circumstances, freeze–thaw cycling constantly reduces BFRC crack resistance, allowing for rapid crack propagation under dynamic stress. Furthermore, the addition of a shear component makes the fracture propagation process more sensitive to the material’s intrinsic heterogeneity, as seen by larger swings in the reported crack propagation velocity.

(3) Crack Propagation Velocity Analysis in Mixed-Mode II Prefabricated Cracks

**Figure 11:**
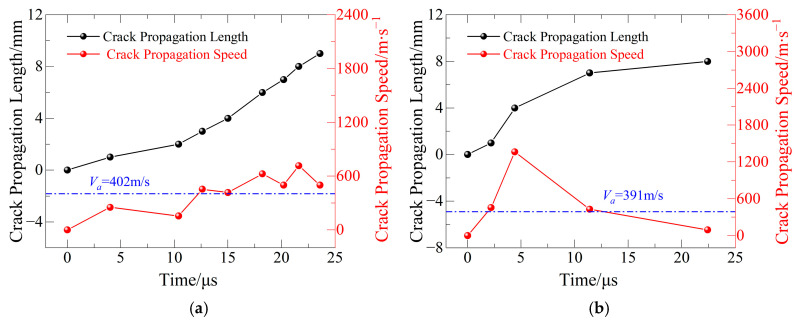

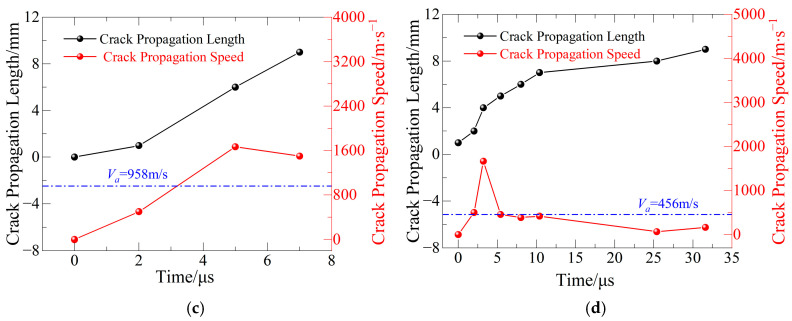
Relationship between crack propagation length/velocity and time for Mode II specimens: (**a**) FTCs 0; (**b**) FTCs 10; (**c**) FTCs 20; and (**d**) FTCs 30.

The fluctuation of crack tip placements and crack propagation velocities of basalt fiber-reinforced concrete (BFRC) specimens under various freeze–thaw cycle counts at a loading angle equivalent to pure Mode II fracture is depicted in Figure 9. In contrast to Mode I fractures, cracks at this loading angle primarily start and spread under a shear-dominated stress environment, and their propagation routes are more convoluted and intricate, as shown in Figure 11; the minimum, maximum, and average propagation velocities are shown in Table 8.

According to the experimental findings, the fracture propagation velocity progressively rises with the number of cycles for the first three groups of freeze–thaw cycles. This suggests that the shear fracture resistance of BFRC is considerably weakened by freeze–thaw cycles. Under shear-dominated stress conditions, the material is particularly vulnerable to fast fracture propagation due to the growth of internal microcracks and the weakening of interfaces caused by freeze–thaw activity.

On the other hand, because the fracture propagation gauge (CPG) only collected a small number of data points, the average fracture propagation velocity for the fourth group seems to be lower. The main cause is the extremely unpredictable crack propagation path under pure Mode II fracture; as cracks propagate tortuously, they progressively stray from the prefabricated crack line and eventually leave the CPG’s effective measurement area, making it impossible to continuously record the crack progression. This event emphasizes even more how sensitive Mode II crack propagation behavior is to the applied stress conditions and the heterogeneous nature of the material.

#### 3.2.2. Analysis of Crack Propagation Velocity Under Static Loading

**Figure 12:**
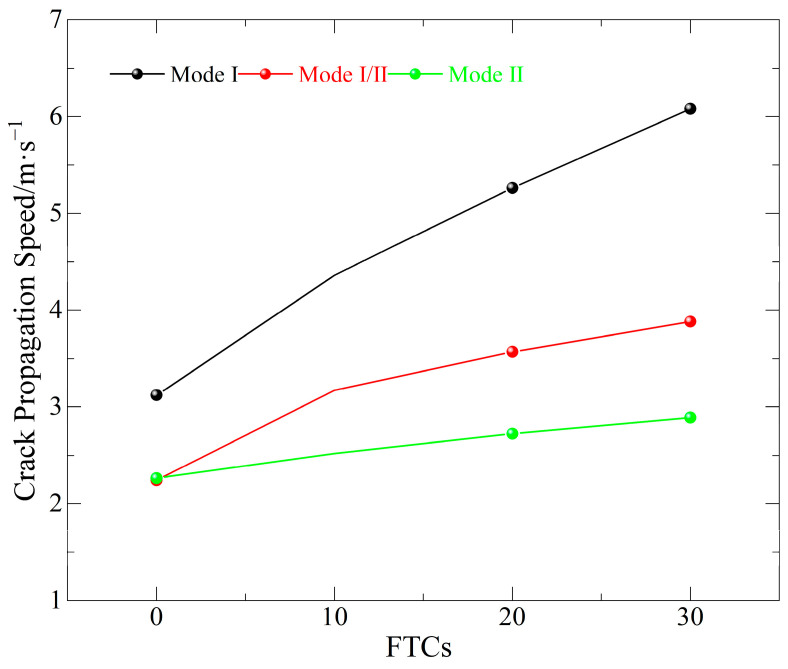
Relationship between crack propagation velocity and number of freeze–thaw cycles under static loading.

The average crack propagation velocities of basalt fiber-reinforced concrete (BFRC) specimens exposed to varying numbers of freeze–thaw cycles at loading angles (θ=0°, 15° and 28.89°) corresponding to pure Mode I, Mode I/II mixed, and pure Mode II fractures are summarized as shown in Figure 12.

In general, the externally applied loads under static loading circumstances are significantly less than dynamic impact loads, leading to a comparatively steady fracture propagation process. The average crack velocity increases with the number of freeze–thaw cycles in an essentially linear fashion, and the variations in fracture velocity over time are much diminished. This suggests that under quasi-static circumstances, the fracture propagation behavior more closely reflects the deterioration brought on by freeze–thaw cycles.

Further study reveals that as the number of freeze–thaw cycles grows, so does the average crack propagation velocity for all three fracture modes, demonstrating a continuing decrease in BFRC’s overall crack resistance. There are significant variations in the fracture modes: cracks propagate fastest under pure Mode I circumstances, followed by Mode I/II mixed fractures, and slowest under Mode II conditions. This finding shows that, under the identical freeze–thaw conditions, tensile-dominated fracture modes promote quicker crack formation, whereas shear-dominated modes provide more resistance to crack propagation.

### 3.3. Analysis of the Effect of Fracture Mode on Crack Propagation Speed Under Dynamic and Static Loads

**Figure 13:**
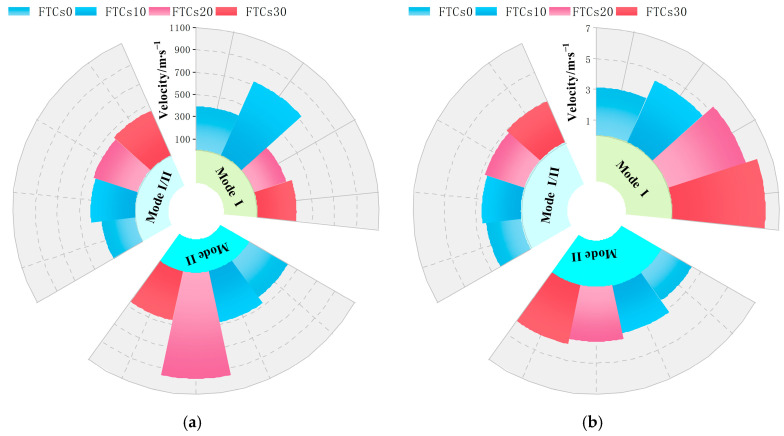
Relationship between crack propagation velocity and freeze–thaw cycles: (**a**) Dynamic crack propagation velocity; and (**b**) static crack propagation velocity.

The connection between the number of freeze–thaw cycles and the crack propagation speed for basalt fiber-reinforced concrete (BFRC) specimens under dynamic and static loading conditions, as shown in Figure 13a and Figure 13b, respectively. It is evident that the fracture propagation speed clearly oscillates as the number of freeze–thaw cycles increases, independent of the prefabricated crack inclination. This suggests that the fracture behavior of BFRC is subject to a continually increasing deterioration impact due to freeze–thaw cycling. Internal pores enlarge, interfacial weakening takes place, and many microcracks are created and linked as the number of freeze–thaw cycles rises. The barrier to crack growth dramatically diminishes when the main fracture interacts with or coalesces with these microcracks during propagation, leading to stage-wise fast increases in crack propagation speed.

When the magnitudes of fracture propagation speeds under different loading situations are compared, the static loading crack speed is about a tenth of that under dynamic loading, indicating a clear order-of-magnitude difference. This difference is mostly due to the dynamic impact’s short duration and high loading rate, which transfers significant energy to the fracture front in a very short period of time, resulting in fast crack propagation. In contrast, static loading levels are significantly lower, resulting in a smoother fracture propagation process. Under static loading, fracture speed changes are lower as the number of freeze–thaw cycles increases, and the overall trend seems more linear. This smoother response partially covers the variations between fracture modes, resulting in less noticeable changes in crack propagation speed under dynamic stress between the three fracture modes. Further analysis of the various fracture modes under static stress revealed that pure Mode I fracture specimens had a substantially faster crack propagation rate than I/II mixed-mode and pure Mode II specimens. This discovery is consistent with the normal fracture characteristics of brittle materials. Brittle materials, such as concrete, have substantially lower tensile strength than shear strength. As a result, in a tensile-dominated stress state, opening-mode crack propagation is more likely, which explains why Mode I fractures propagate faster and have a stronger dominance under static loading circumstances.

## 4. Analysis of Dynamic and Static Fracture Processes with Various Fiber Contents

### 4.1. BFRC Model Development and Parameters

#### 4.1.1. Model Development

A three-dimensional finite element model of the central straight-crack platform Brazilian disk (CSTFBD) specimen was created with the general-purpose finite element program ANSYS. The pre-processing work was done in the ANSYS environment. The concrete matrix was discretized using Solid164 solid elements of Φ50 mm × 25 mm. A central straight crack, 1 mm wide and 10 mm long, was pre-inserted in the center of the specimen, and the crack tip was sharpened to more accurately capture the stress concentration effects at the crack front. The general shape, crack arrangement, and dimensional parameters of the specimen were kept constant with the physical investigations to ensure that numerical simulation and experimental findings were comparable.

Beam elements were employed to discretize the basalt fibers and imitate their reinforcing function inside the concrete matrix. A bespoke fiber command script was used to scatter the fibers randomly throughout the specimen, mirroring the non-uniform dispersion of fibers in the genuine material. The meshing of the concrete specimen was done with ANSYS’ integrated ICEM CFD module. Hexahedral elements were used throughout the model to increase computational accuracy and numerical stability. The global mesh size was set to 0.6 mm, and local mesh refinement was used in the crack tip region to reduce the element size to 0.2 mm. This provides an accurate capture of stress field development during fracture initiation and propagation.

**Figure 14:**
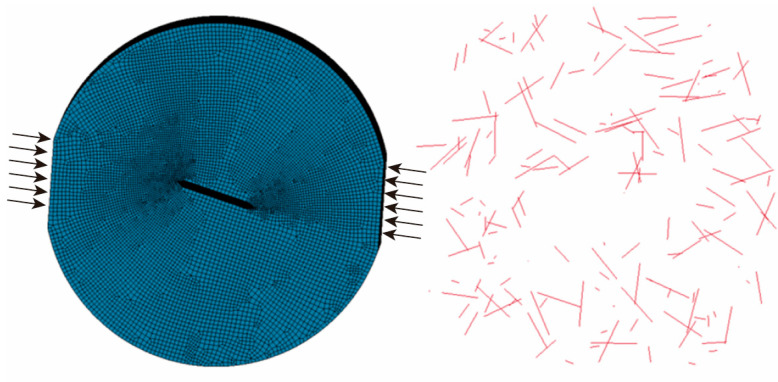
Concrete and basalt fiber model.

The completed 3D finite element model of the concrete matrix with embedded basalt fibers is shown in Figure 14. This modeling strategy balances computational efficiency with detailed representation of the crack tip region, providing a reliable foundation for subsequent numerical simulation and analysis of dynamic and static fracture processes.

#### 4.1.2. Constitutive Model and Parameters of Basalt Fiber-Reinforced Concrete

In this study, the elastic behavior of basalt fiber-reinforced concrete was described using the *MAT_ELASTIC (Material Type 1) model. This model is based on the isotropic linear elastic assumption, with constitutive relations defined by the generalized Hooke’s law:

(6) $$\sigma_{ij}=2G\varepsilon_{ij}+\lambda \varepsilon_{kk}\delta_{ij}$$

where G is the shear modulus, λ is the Lamé constant, and $\delta_{ij}$ is the Kronecker symbol. Since the *MAT_ELASTIC model itself does not incorporate failure criteria and cannot simulate cracking or element deletion after reaching the ultimate strength, the *MAT_ADD_EROSION keyword was employed to supplement the elastic material with a tensile strength failure criterion.

To properly replicate the failure behavior of the center straight-through flattened Brazilian disk (CSTFBD) specimens during loading, the numerical model must include an appropriate failure criterion that represents crack initiation and propagation. In this study, the *MAT_ADD_EROSION keyword is utilized in the LS-DYNA framework to specify element failure, with the highest tensile stress used as the failure criteria. When the major tensile stress in an element reaches or exceeds the stated ultimate tensile stress, the element is deemed failed and removed from the computation. This method enables autonomous crack initiation, propagation, and penetration in the numerical model.

This failure model accurately depicts the fracture properties of brittle materials like concrete under tensile-dominated circumstances and is consistent with the crack propagation patterns seen in the experiments. When combined with the previously applied mesh refinement at the crack tip and the defined material parameters, this method accurately captures the stress release and damage evolution during crack propagation in BFRC under various freeze–thaw cycles, providing a reliable numerical basis for subsequent fracture mechanism analysis.

**Figure 15:**
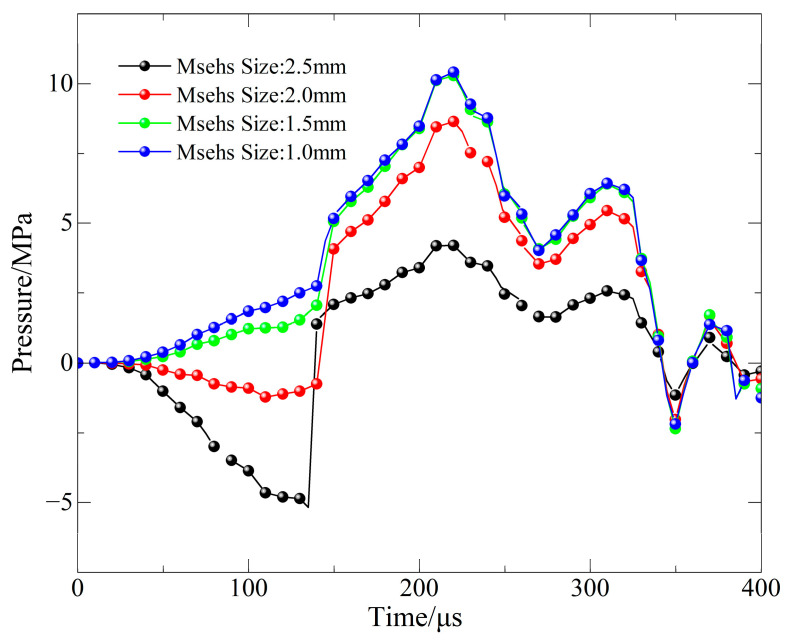
Mesh convergence verification curves.

The rationality of the adopted mesh was verified by comparing simulation results obtained with different mesh sizes against experimental data. As shown in Figure 15, the pressure curves from simulations using mesh sizes of 1.0 mm, 1.5 mm, 2.0 mm, and 2.5 mm are plotted alongside the experimental curve for comparison. A consistent mesh size of 1 mm was employed throughout all simulations in this study.

### 4.2. Simulation of Fracture Process Under Different Crack Modes

**Figure 16:**
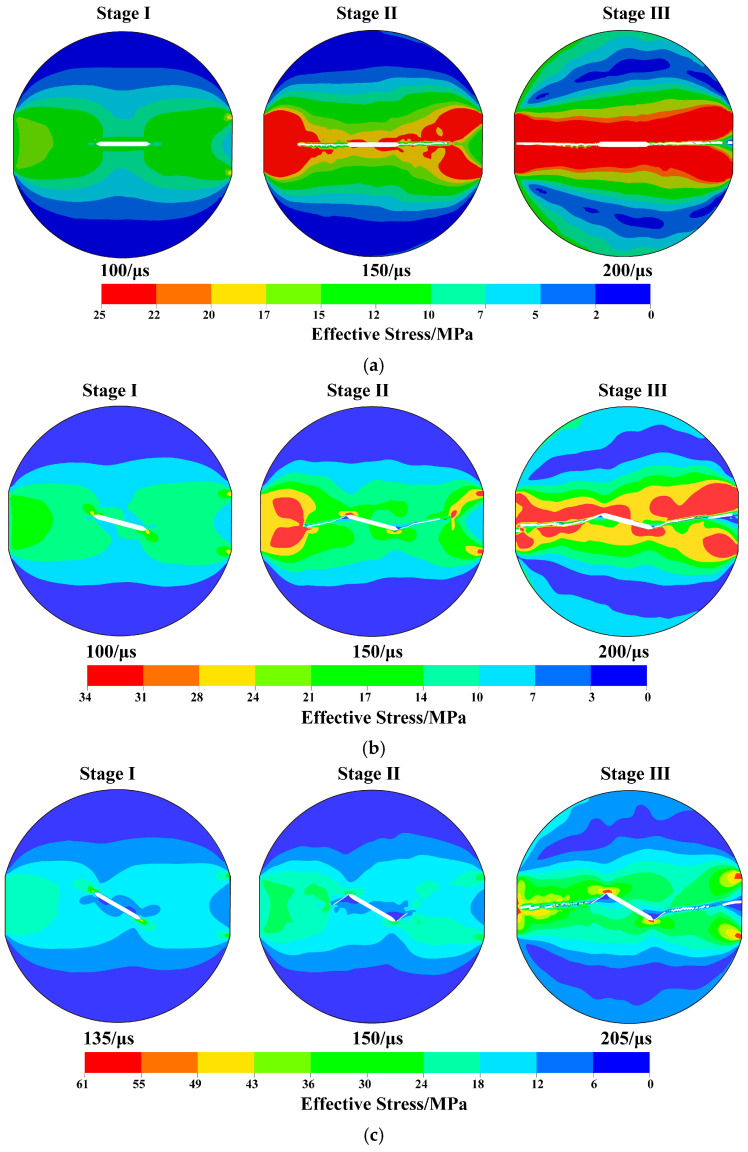
Evolution of the effective stress field in specimens under different fracture modes: (**a**) Evolution of the effective stress field in Mode I specimen; (**b**) evolution of the effective stress field in Mode I/II mixed-mode specimen; and (**c**) evolution of the effective stress field in Mode II specimen.

The development of the effective stress field in center straight-through flattened Brazilian disk (CSTFBD) specimens with three distinct pre-crack inclination angles without freeze–thaw cycles is shown in Figure 16. Here, the effective stress is defined as the Von Mises equivalent stress, $\sigma_{\mathrm{eff}}=\sqrt{\frac{1}{2}[{\left(\sigma_{1}-\sigma_{2}\right)}^{2}+{\left(\sigma_{2}-\sigma_{3}\right)}^{2}+{\left(\sigma_{3}-\sigma_{1}\right)}^{2}]}$, which characterizes the overall stress level under multiaxial loading. It is evident that the effective stress in the specimens is mostly concentrated at the fracture tip during the initial loading stage, independent of the pre-crack inclination. This suggests that the crack tip is constantly the dominating stress concentration zone.

The fracture starts at the tip and progressively moves forward as the external stress increases. The related effective stress field changes along the fracture route as its propagation path gradually deflects in the orientations of the side platforms. The effective stress dispersed along the fracture progressively moves in the direction of the disk edge areas on both sides of the platforms during crack propagation, where it re-concentrates. The highest effective stress inside the specimen is now focused at the disk edges rather than at the fracture tip when the crack eventually penetrates the whole specimen. Following fracture penetration, the border areas become the new stress concentration zones, reflecting the redistribution of stress within the entire load-bearing system.

Additionally, it is evident from the maximum primary stress distribution in Figure 13 that the total effective stress level inside the specimen rises with the pre-crack inclination angle at the point where the fracture completely enters the specimen. This suggests that the stress distribution and fracture history of CSTFBD specimens are strongly influenced by the pre-crack inclination: the greater the crack inclination, the higher the stress level needed for failure, and consequently, the more difficult it is for the crack to spread.

**Figure 17:**
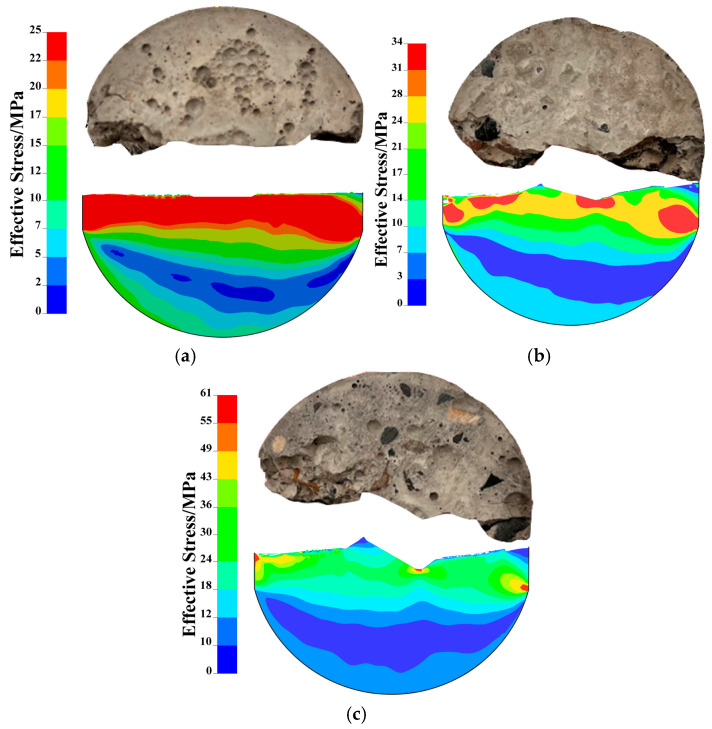
Comparison of failure morphology numbers for specimens under different fracture modes: (**a**) Failure morphology of Mode I specimen; (**b**) failure morphology of Mode I/II mixed-mode specimen; and (**c**) failure morphology of Mode II specimen.

The numerical simulation findings and actual fracture patterns of CSTFBD specimens with three distinct pre-crack inclination angles are contrasted in Figure 17. It is evident that the ultimate failure morphologies and crack propagation routes derived from the numerical simulations are in agreement with the experimental findings. Both exhibit radial tensile-splitting fracture patterns along the disk, suggesting that the fundamental fracture modes of basalt fiber-reinforced concrete specimens under dynamic loading circumstances may be properly reproduced by the constructed computational model and failure criterion.

The experimentally observed fracture patterns reveal discrete triangular crushing zones in the platform loading regions on both sides of the specimen, with the triangular region on the incident bar side (left end of the specimen) being much bigger than that on the opposite side. This asymmetric failure represents the inertial effects and stress non-uniformity caused by wave incidence, reflection, and transmission during the SHPB test, which have a significant impact on local fracture morphology.

In contrast, the numerical simulation findings do not properly depict the platform areas’ triangle crushing zones. The predicted fracture pattern seems rather symmetric, with weaker local crushing. This mismatch is mostly due to the present simulation’s failure to explicitly describe the Hopkinson pressure bars in order to minimize computing scale and save calculation time, consequently ignoring the inertial effects and stress wave propagation characteristics that occur during bar–specimen interactions. Nonetheless, the simulation results agree well with experimental observations in key areas such as crack initiation locations, propagation paths, and overall failure mode, indicating that the established numerical model is reliable and applicable to macroscopic fracture behavior.

## 5. Discussion

### 5.1. The Effect of Fiber Content on the Fracture Process

**Figure 18:**
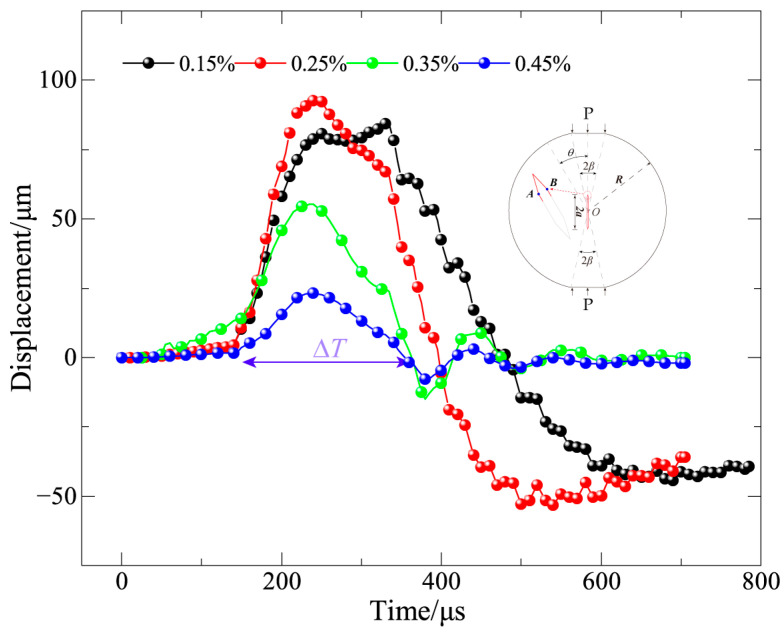
Crack tip opening displacement curve.

The crack-tip opening displacements (CTOD) [39,40,41,42] at endpoints A and B of Mode I fracture specimens under various basalt fiber contents are shown numerically in Figure 18. The CTOD time–history curves show a distinct and consistent trend as the basalt fiber content rises: the peak opening displacement gradually decreases, and the crack opening duration gradually shortens, suggesting that the addition of basalt fibers successfully inhibits crack-tip deformation.

According to the time–history response, there is a discernible crack-tip opening at locations A and B around 40 μs after the external stress is applied, indicating the beginning of fast fracture initiation and propagation. The time to attain the peak crack-tip opening displacement advances considerably with increasing fiber content, occurring at 380 μs, 238 μs, 228 μs, and 243 μs for the corresponding fiber content levels. This shows that the material’s energy dissipation and confinement capability under dynamic loading are improved by a larger basalt fiber content, which results in a more controlled crack propagation process, a shorter period of fast fracture opening, and a smaller maximum opening displacement.

Overall, the observed decrease in crack-tip opening displacement with increasing fiber content directly reflects the pullout energy-dissipation and crack-bridging effects of basalt fibers in the concrete matrix, which help to improve the material’s dynamic fracture resistance and delay the spread of cracks.

### 5.2. Analysis of Crack Propagation Path

**Figure 19:**
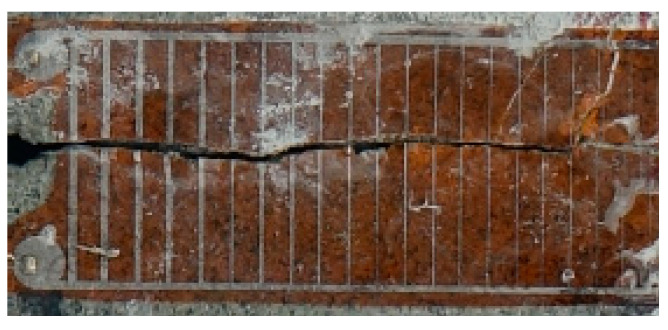
Crack propagation path.

The real fracture propagation routes in specimens of basalt fiber-reinforced concrete (BFRC) are depicted in Figure 19. The entire course deviates from an ideal straight line, and it is evident that the fractures display prominent tortuous and meandering features during propagation. A closer look at the crack propagation velocity results from Figure 9, Figure 10 and Figure 11 shows that, in most cases, there are comparatively long intervals between adjacent CPG (crack propagation gauge) resistance wire ruptures—for instance, between the third and fourth wires in Figure 9c and the sixth and seventh wires in Figure 10d. This shows that rather than growing steadily during the extension phase, the fracture propagation velocity varies greatly.

The underlying causes of the uneven crack propagation speed and convoluted course may be explained by two major aspects:

(1) This is a prevalent feature of brittle materials under impact loading. When fragile materials are exposed to high-speed impact, fractures spread quickly once they begin. However, as the fracture propagation velocity reaches a critical threshold, dynamic instability near the crack tip rises, resulting in significant oscillations in propagation speed. Furthermore, small parabolic or groove-like structures frequently appear along the fracture route, and these local unstable propagation processes cause further deviations and abnormalities in the crack trajectory.
(2) It is intimately linked to the heterogeneous structure of concrete itself. Concrete comprises coarse and fine materials that are scattered randomly, as well as many microcracks and voids. When a primary crack enters a location with existing microcracks, fast coalescence can occur, resulting in a transient increase in propagation speed. When a fracture travels through a coarse aggregate, the higher strength of the aggregate inhibits crack development, dramatically lowering propagation velocity. If a fracture spreads over the interface between coarse aggregate and mortar, it may deform or split. The alternating activity of these processes causes significant changes in propagation speed and convoluted pathways in heterogeneous brittle materials such as concrete.

### 5.3. Mechanistic Discussion of Fiber–Matrix Interaction Under Freeze–Thaw Cycles

The degradation of fiber-reinforced concrete under freeze–thaw cycles (FTC) is fundamentally governed by the progressive deterioration of fiber–matrix interfacial properties. Based on the experimental observations and microstructural analysis, the following mechanistic framework is proposed to elucidate the fiber–matrix interaction under FTC conditions.

#### 5.3.1. Interfacial Damage Accumulation

**Figure 20:**
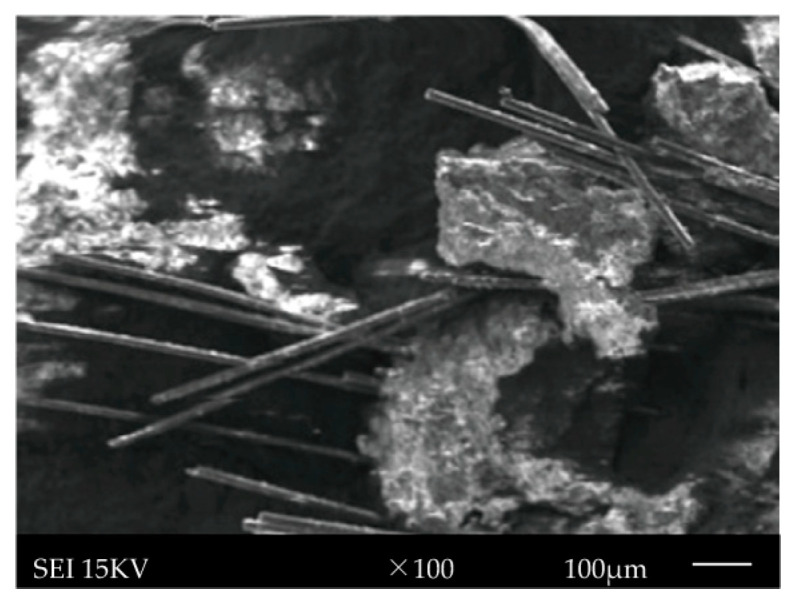
SEM images of basalt fiber-reinforced concrete microstructure.

During FTC, the cyclic phase transformation of water within capillary pores generates repetitive frost heave pressures. These pressures concentrate at the fiber–matrix interfacial transition zone (ITZ), which is inherently more porous and weaker than the bulk matrix. As FTC progresses, microcracks initiate at the ITZ and propagate along the fiber–matrix interface. This is evidenced by SEM observations, as shown in Figure 20, which reveal a widening of interfacial gaps and exfoliation of hydration products from fiber surfaces after exposure to FTC. The interfacial shear strength (IFSS) progressively degrades with increasing FTCs, reducing the load transfer efficiency between matrix and fibers.

#### 5.3.2. Evolution of Fiber-Bridging Mechanisms

The fiber-bridging effect, which is critical for controlling crack propagation, undergoes a significant transformation during FTC. In the initial stage (0 cycles), fibers remain well-bonded to the matrix, and crack bridging is governed by elastic stress transfer across the interface. As interfacial damage accumulates (10~20 cycles), the bridging mechanism transitions from elastic stress transfer to frictional pullout, characterized by partial debonding and sliding of fibers within the matrix. At advanced FTC stages (>20 cycles), extensive interfacial degradation leads to premature fiber pullout with reduced energy dissipation, diminishing the composite’s crack resistance.

#### 5.3.3. Fracture Mode Transition

The progressive interfacial damage induces a systematic transition in fracture behavior. Analysis of post-FTC fracture surfaces, as shown in Figure 20, reveals that specimens subjected to fewer FTCs predominantly exhibit fiber pullout with rough fiber surfaces, indicating strong interfacial bonding, which is consistent with the findings reported in [3,18]. With increasing FTC cycles, fracture surfaces show increasingly clean fibers with smooth surfaces, suggesting interfacial debonding as the dominant failure mode. This transition from cohesive (fiber rupture) to adhesive (interfacial debonding) failure reflects the gradual deterioration of fiber–matrix interfacial integrity.

## 6. Conclusions

This work comprehensively studied the dynamic and static fracture behavior of basalt fiber-reinforced concrete (BFRC) utilizing center straight-crack platform Brazilian disk (CSTFBD) specimens with different prefabricated crack inclinations during freeze–thaw cycles. The influence of freeze–thaw cycle number, prefabricated crack inclination, and basalt fiber content on fracture propagation behavior was investigated. In addition, ANSYS/LS-DYNA numerical simulations were used to investigate the development of the maximum primary stress field during crack propagation. The key findings are as follows:

(1) Freeze–thaw cycles dramatically speed up fracture growth. As the number of freeze–thaw cycles grew, the average fracture propagation velocity in BFRC exhibited an overall oscillatory development pattern, with crack pathways gradually changing from relatively straight to convoluted. The crack propagation velocity rose from 305.81 m·s^−1^ to 494.28 m·s^−1^ during dynamic loading and from 2.24 m·s^−1^ to 6.08 m·s^−1^ under static loading, suggesting that freeze–thaw degradation severely impairs the material’s dynamic fracture behavior.
(2) Crack propagation velocity is mostly determined by the loading rate. The fracture propagation velocity of CSTFBD specimens was much greater under dynamic loading than static loading; the average crack propagation velocity ranges from 263 m/s to 726 m/s, with a difference of around two orders of magnitude. Under static stress, pure Mode I fracture specimens had much faster crack propagation velocity than Mode I/II mixed and pure Mode II fractures, indicating that brittle materials are more prone to opening-mode fractures under tension-dominated circumstances. Under dynamic stress, the changes in crack propagation velocity between fracture modes were not significant.
(3) The prefabricated crack inclination has a significant impact on the evolution of the stress field and the failure characteristics. The effective stress in the specimen ranged from approximately 25 MPa to 61 MPa, depending on the crack inclination angle. For all three crack orientations, the effective stress was initially localized at the constructed fracture tip and subsequently moved to the platform sides as the crack spread. Once the break had entered the specimen, the most effective stress was concentrated along the borders of the disk platforms. Furthermore, as the crack inclination grew, so did the total effective stress level in the specimen, demonstrating that crack inclination had a significant impact on fracture resistance and failure mode.
(4) Numerical models accurately represent experimental fracture properties. The fracture propagation pathways, stress concentration sites, and overall failure modes reported from simulations were consistent with experimental results, confirming the validity of the numerical model and material characteristics. The simulation findings also showed the stress evolution process of crack propagation under the combined impacts of freeze–thaw damage and fracture inclination, providing a theoretical foundation for the safety evaluation and design of shotcrete structures in cold-region excavation projects.
(5) Future work should: (i) explore multiaxial fracture behavior; (ii) conduct longer-term freeze–thaw tests for durability modeling; and (iii) employ advanced microscopy for quantitative interfacial damage characterization.

## Figures and Tables

**Table 1:** Basic physical performance indicators of basalt fiber [31].

Length/mm	Diameter/μm	Density/(kg·m^−3^)	Elastic Modulus/GPa	Elongation at Break/%	Tensile Strength/MPa
6	15	2600	95~115	2.4~3.1	3300~4500

**Table 2:** Mix proportion of basalt fiber-reinforced concrete [31].

Water/ (kg·m^−3^)	Cement/ (kg·m^−3^)	Sand/ (kg·m^−3^)	Stone/ (kg·m^−3^)	Basalt Fiber/ (kg·m^−3^)
184	368	718	1122	6

**Table 3:** Test conditions for static fracture characteristics of BFRC.

Number	Freeze–Thaw Cycles	Prefabricated Crack Inclination Angle/°	Loading Rate/mm·min^−1^
S-0-0	0	0	0.5
S-10-0	10	0	0.5
S-20-0	20	0	0.5
S-30-0	30	0	0.5
S-0-15	0	15	0.5
S-10-15	10	15	0.5
S-20-15	20	15	0.5
S-30-15	30	15	0.5
S-0-28.89	0	28.89	0.5
S-10-28.89	10	28.89	0.5
S-20-28.89	20	28.89	0.5
S-30-28.89	30	28.89	0.5

Note: In the numbering system, the first symbol indicates static loading, the second digit represents the number of freeze–thaw cycles, and the third digit represents the prefabricated crack inclination angle.

**Table 4:** Test conditions for dynamic fracture characteristics of BFRC.

Number	Freeze–Thaw Cycles	Prefabricated Crack Inclination Angle/°	Impact Velocity/m·s^−1^
D-0-0	0	0	5
D-10-0	10	0	5
D-20-0	20	0	5
D-30-0	30	0	5
D-0-15	0	15	5
D-10-15	10	15	5
D-20-15	20	15	5
D-30-15	30	15	5
D-0-28.89	0	28.89	5
D-10-28.89	10	28.89	5
D-20-28.89	20	28.89	5
D-30-28.89	30	28.89	5

Note: In the numbering system, the first symbol indicates dynamic loading, the second digit represents the number of freeze–thaw cycles, and the third digit represents the prefabricated crack inclination angle.

**Table 5:** Material parameters of BFRC under different numbers of freeze–thaw cycles.

Freeze–Thaw Cycles	Density/kg·m^−3^	Elastic Modulus/GPa	Poisson’s Ratio
0	2400	19.326	0.201
10	2400	19.049	0.196
20	2400	17.982	0.195
30	2400	16.662	0.194

**Table 6:** The crack propagation velocity under different freeze–thaw cycles (**Mode I**).

Freeze–Thaw Cycles	Min. Crack Velocity/m·s^−1^	Max. Crack Velocity/m·s^−1^	Avg. Crack Velocity/m·s^−1^
0	64	1000	263
10	81	1667	726
20	145	455	263
30	200	1000	355

**Table 7:** The crack propagation velocity under different freeze–thaw cycles (**Mode I/II**).

Freeze–Thaw Cycles	Min. Crack Velocity/m·s^−1^	Max. Crack Velocity/m·s^−1^	Avg. Crack Velocity/m·s^−1^
0	222	714	303
10	114	555	397
20	294	625	418
30	74	625	402

**Table 8:** The crack propagation velocity under different freeze–thaw cycles (**Mode II**).

Freeze–Thaw Cycles	Min. Crack Velocity/m·s^−1^	Max. Crack Velocity/m·s^−1^	Avg. Crack Velocity/m·s^−1^
0	156	714	402
10	91	1363	391
20	500	1667	958
30	67	1667	456

## Data Availability

The original contributions presented in this study are included in the article. Further inquiries can be directed to the corresponding author.
